# Therapeutic effect of platelet-rich plasma on glucocorticoid-induced rat bone marrow mesenchymal stem cells *in vitro*

**DOI:** 10.1186/s12891-022-05094-2

**Published:** 2022-02-15

**Authors:** Yanxue Wang, Shuo Luan, Ze Yuan, Caina Lin, Shengnuo Fan, Shaoling Wang, Chao Ma, Shaoling Wu

**Affiliations:** Department of Rehabilitation Medicine, Sun Yat-sen Memorial Hospital, Sun Yat-sen University, Guangzhou, 510030 Guangdong China

**Keywords:** Platelet-rich plasma, Glucocorticoid, Bone marrow mesenchymal stem cells

## Abstract

**Background:**

Glucocorticoid-induced osteonecrosis of the femoral head (GIONFH) is a progressive and disabling disease caused by long-term or high-dose glucocorticoid use. Decreased osteogenesis and proliferation of bone marrow mesenchymal stem cells (BMSCs) are the main pathogenesis of GIONFH. Platelet-rich plasma (PRP) has been shown to play a promising role in bone regeneration. However, the effects of PRP on glucocorticoid-induced BMSCs inhibition remains elusive. The objective of this study was to explore whether PRP could improve the *in vitro* biological activities of BMSCs inhibited by high-dose glucocorticoid *in vitro*.

**Methods:**

In this study, a dexamethasone (Dex)-induced in vitro cell model was established. The effects of PRP on proliferation, migration, cell cycle and apoptosis of rat BMSCs induced with high-dose Dex compared to BMSC_CTRL_, using CCK-8 assay, transwell, flow cytometry and TUNEL assay, respectively. We further performed the alkaline phosphatase (ALP) and alizarin red (ALR) staining to explore the influence of PRP on osteogenic differentiation. Western Blot was used to detect the expression of Bcl-2, Caspase-3, RUNX2 apoptosis, and osteogenic-related proteins.

**Results:**

We observed increased apoptosis rate and Caspase-3 expression, and the decreased migration and osteogenic differentiation, and down-regulation of RUNX-2 and Bcl-2 expression in Dex-induced BMSCs. PRP could reverse these inhibitory effects of Dex, and enhance the BMSCs proliferation, migration, and osteogenic ability *in vitro.*

**Conclusion:**

Our *vitro* study showed that PRP significantly protected BMSCs from Dex-induced apoptosis, and further promoted BMSCs proliferation, migration, and osteogenic differentiation. This study provides a scientific basis for the prevention and treatment of GIONFH with PRP. Meanwhile, it also lays the foundation for the application of PRP in other musculoskeletal diseases.

**Supplementary Information:**

The online version contains supplementary material available at 10.1186/s12891-022-05094-2.

## Background

Glucocorticoid-induced osteonecrosis of the femoral head (GIONFH) is a common disease after long-term or high-dose glucocorticoid use. Generally, the incidence of GIONFH is related to dose and administration routes of glucocorticoid, even though there is no available exact definition of dose and duration of use. In addition, the individual susceptibility varies in different diseases. Based on existing clinical data,, cumulative methylprednisolone (> 2 g for more than three months) significantly increased the risk for GIONFH [1]. As ONFH progresses, the femoral head eventually collapses, resulting in pain and dysfunction [2–4]. Studies have shown that high-dose of glucocorticoid decreases the proliferation and osteogenic capacity and increases the apoptosis of bone marrow mesenchymal stem cells (BMSCs), which aggravates femoral head necrosis [5–7]. On the onset of this disease, the number and activity of BMSCs in the femoral head, neck, and metaphyseal regions are reduced, leading to the limitation of the body’s self-repair ability, and the gradual progression to femoral head necrosis [8, 9]. Thus, the dysfunction of BMSCs has been regarded as important pathogenesis of GIONFH [6, 10]. Therefore, the strategies that promote proliferation and differentiation, and inhibit apoptosis of BMSCs at an early stage may significantly trigger the repair process and prevent the progression of GIONFH [11–13].

Platelet-rich plasma (PRP) is a concentrated platelet prepared by centrifugation of autologous whole blood, and PRP releases a variety of growth factors, including transforming growth factor-β1 (TGF-β1), vascular endothelial growth factor (VEGF), platelet-derived growth factor (PDGF), and insulin-like growth factor (IGF), basic fibroblast growth factor (b-FGF) [14]. These growth factors have been proved to stimulate the proliferation and differentiation of stem cells and promote the repair of bone tissues [15–18]. However, the effect of PRP on glucocorticoid-induced BMSCs remains elusive.

The purpose of this study was to explore whether PRP could improve the inhibited biological activities of BMSCs induced by high-dose of glucocorticoid. Our results provide important insights into the potential of PRP and its mechanisms in influencing BMSCs activities *in vitro*. Therefore, it provides a theoretical basis for the future clinical exploration of PRP in the treatment of ONFH.

## Methods

### Study design and ethics statement

All procedures were carried out according to the guide for the Care and Use of Laboratory Animals and were approved by the Institutional Animal Care and Use Committee of Sun Yat-sen University. Male Sprague-Dawley (SD) rats were purchased from Sun Yat-sen University Animal Experimental Center (Guangzhou, China). Rats were maintained under standard laboratory conditions, with free access to food and water, and housed prior to experiments in an animal room under standard conditions (23 ± 2 °C; 60 ± 10% humidity; 12 h light/dark cycle).

#### Preparation and composition analysis of platelet-rich plasma

Ten 12-week-old SD rats were anesthetized by intraperitoneal injection of 2.5% pentobarbital sodium (40–45 mg/kg). The preparation of PRP was strictly aseptic. Ten milliliter whole blood was drawn from each rat via intracardiac puncture. A total of 9 ml of blood sample was drawn from each rat into 10-ml syringes containing 1 ml sodium citrate anticoagulant, of which 0.5 mL was used for blood count (quantitative analysis of platelet and leukocyte counts). The remaining 9.5 ml was transferred into a sterile test tube and centrifuged twice to obtain fresh PRP [19]. After the first centrifugation (400 g, 10 min), the blood was divided into three layers, with the plasma in the supernatant, a buffy coat layer in the middle, and red blood cells at the bottom. Then, the supernatant was pipetted into another sterile test tube for second centrifugation (800 g, 10 min). The 3/4 upper fraction of the plasma (platelet-poor plasma) was discarded, and the remaining liquid was platelet-rich plasma (about 1.5 mL). 10% CaCl_2_ (C5670, Sigma-Aldrich) plus 1000 U/ml thrombin from bovine plasma (T8021, Solarbio, Beijing, China) was used for activation of the alpha granules in platelets and was added into the apheresis platelets at a volume ratio of 1:10. The activated PRP-containing supernatant was collected and filtered through 0.22-μm aseptic membrane filter and then aliquoted and stored at − 80 °C to avoid repeated freezing and thawing. A 0.2 mL sample of PRP was reserved for the detection of the growth factors.

As mentioned above, the platelets and leukocyte concentrations in the samples of whole blood (0.5 mL) and PRP were measured with the Mindray BC-5000Vet analyzer. ELISA kits were utilized to quantify the concentrations of rat transforming growth factor-beta (TGF-β, LER822–1, Laizee, Shanghai, China) based on the manufacturer’s protocol.

### BMSCs isolation and culture

To investigate the effects of PRP, the BMSCs were induced by Dex (D4902, Sigma-Aldrich). Different interventions were used to assess a series of functional assays, including the control group, the Dex group, and the Dex + PRP group. In cell proliferation assay, migration assay, and osteogenic differentiation assay, the concentration of Dex was 10 μM. The concentration of Dex was 100 μM in the apoptosis experiment.

Bone marrow stem cells (BMSCs) of 4-week-old SD rats were obtained from the femur and tibia according to the method described by Shen [6] and were cultured with complete medium:Dulbecco’s modified Eagle’s medium (DMEM) (Gibco, MA, USA) containing 10% fetal bovine serum (FBS, Gibco), 1% penicillin-streptomycin (Gibco) in an incubator at 37 °C and 5% CO_2_. After 24 h, the medium was changed and the attached cells were washed by phosphate-buffered saline (PBS, Gibco). The culture medium was refreshed every 2 days until adherent cells reach 80–90% confluence, then the cells were passaged for three to five passages for all further experiment use.

### Cell proliferation assay

#### Effect of PRP on Dex-induced BMSC proliferation

To determine the optimal PRP concentration that could promote the proliferation of BMSCs, we first carried out a Cell-counting kit 8 (CCK-8) cell proliferation assay. BMSCs were inoculated in 96-well plates with 3 replicate wells in each group of 3 × 10^3^/well. After 24 h of complete adherence, 100 μL complete medium containing 0, 2.5, 5, 10, and 20% PRP was added. The proliferation of BMSC was measured with a CCK-8 assay kit (K1018–1, ApexBio, MA, USA) at 24, 48, and 72 h. The optimum PRP concentration obtained in this experiment was used in all subsequent experiments.

The CCK-8 kit was used to explore the proliferation rate of BMSCs treated with Dex. A total of 3000/well BMSCs were inoculated into 96-well plates. The cells were divided into three groups: (1) control group, (2) Dex group, and (3) Dex + PRP group. The absorbance was measured at 450 nm using an enzyme-linked immunosorbent assay reader (SpectraMax Plus 384, Molecular Devices, Sunnyvale, CA, USA).

### Flow cytometry

The cell cycle was measured by flow cytometry. The cells were inoculated in a 6-well plate with 1.5 × 10^5^ cells/well. After the cells were completely attached to the wall of the wells, the serum-free medium was incubated overnight, and different interventions were conducted. 2 ml medium was added to each well. After 24 h of induction, the cells were digested by trypsin and fixed overnight with 75% iced ethanol. After being washed by PBS, propidium iodide (PI, C0080, Solarbio, Beijing, China) and RNase A(R8020, Solarbio) were added and then detected by flow cytometer (BD FACSCalibur; BD Biosciences). The results were presented as proliferation index PrI (percentage of cells at S and G2M phase), which was calculated as PrI = (S+ G_2_ /M) / (G _0_ /G1 + S+ G_2_ /M).PrI indicated the proportion of cells in active proliferation [20].

### BMSCs migration assay

BMSCs migration assay was performed by 24-well plates with 8 μm transwell (Corning) upper chambers. 1 × 10^4^/well BMSCs were seeded into the upper chamber, and 600-uL of the complete medium was added into the lower chamber according to the experimental grouping. After incubating for twelve hours, non-migrative cells on the upper surface of the transwell membrane were wiped with a cotton swab. Then the cells on the lower surface of the membrane were fixed with 4% paraformaldehyde for 20 min, stained with 0.5% crystal violet for 15 min and washed with PBS. It was then observed with a light microscope (Nikon NI-U). Five different visual fields were randomly selected and photographed, and the migratory cells were counted by Image J software (National Institutes of Health, Bethesda, MD, USA).

### TUNEL assay

The terminal deoxynucleotidyl transferase (TdT) dUTP nick-end labeling (TUNEL) assay was used to estimate the effect of PRP on BMSCs cell apoptosis according to the manual [21]. After 48 h of culture with the serum-free medium in the presence or absence of PRP, the cells were fixed with 4% paraformaldehyde for 25 min at 37 °C, incubated with 0.3% Triton X-100 for 5 min, and washed with PBS twice between each step. The cells were incubated with TUNEL reagent (C1086, Beyotime, Shanghai, China) according to the manufacturer’s instructions, and the nuclei were counterstained with DAPI (C1002, Beyotime) for five minutes. The samples were observed and imaged under a fluorescence microscope (Olympus IX 71).

### Alkaline Phosphatase (ALP) and Alizarin Red (ALR) staining

Bone marrow mesenchymal stem cells were seeded on a 24-well plate at a density of 1 × 10^5^ cells/well. After 7 days of using osteogenic differentiation induction medium (RAXMX-90021, Cyagen, Guangzhou, China), ALP staining (Beyotime) was performed. 14 days later, alizarin red staining (Cyagen) was performed according to the manufacturer’s protocol. The cells were washed twice with PBS, fixed with 4% paraformaldehyde for half an hour, and then stained. Before taking pictures, each well was washed 3 times with double distilled water.

### Western blotting

The protein was extracted from the cells using radio immunoprecipitation assay (PC101, RIPA) lysis buffer (EpiZyme, Shanghai, China). The protein concentration was determined using Thermo Scientific™ Pierce™ BCA (23227). An equal amount of protein was separated by 10–15% sodium dodecyl sulfate-polyacrylamide gel electrophoresis (SDS-PAGE) and then transferred to a polyvinylidene fluoride (PVDF) membrane (Millipore, Billerica, MA, USA). After blocking with protein-free fast blocking buffer (PS108, EpiZyme), it was incubated with primary antibodies against Runx2 (Affinity, AF5186), Bcl-2 (Affinity, AF6139), Caspase-3 (Huabio, ET1608–64), and GAPDH (EpiZyme, LF205) at 4 °C overnight, and then incubated with the secondary antibody at 37 °C for 1 h. Thereafter, the proteins were visualized using Omni ECL reagent (SQ201, ECL; EpiZyme) under e-Blot (Touch Imager, Shanghai, China). The gray densitometric was analyzed using Image J (USA).

## Statistical analysis

All of these experiments were repeated three times. Data were shown as mean ± standard deviation (SD) and analyzed with the statistical software GraphPad Prism 8 (GraphPad Software, Inc., USA). Means of multiple groups were compared by one-way analysis of variance (ANOVA). *P* < 0.05 was considered statistically significant.

## Results

### PRP alleviated the inhibitory effects of Dex on proliferation and migration of BMSCs while protecting cells from apoptosis

**Fig. 1:**
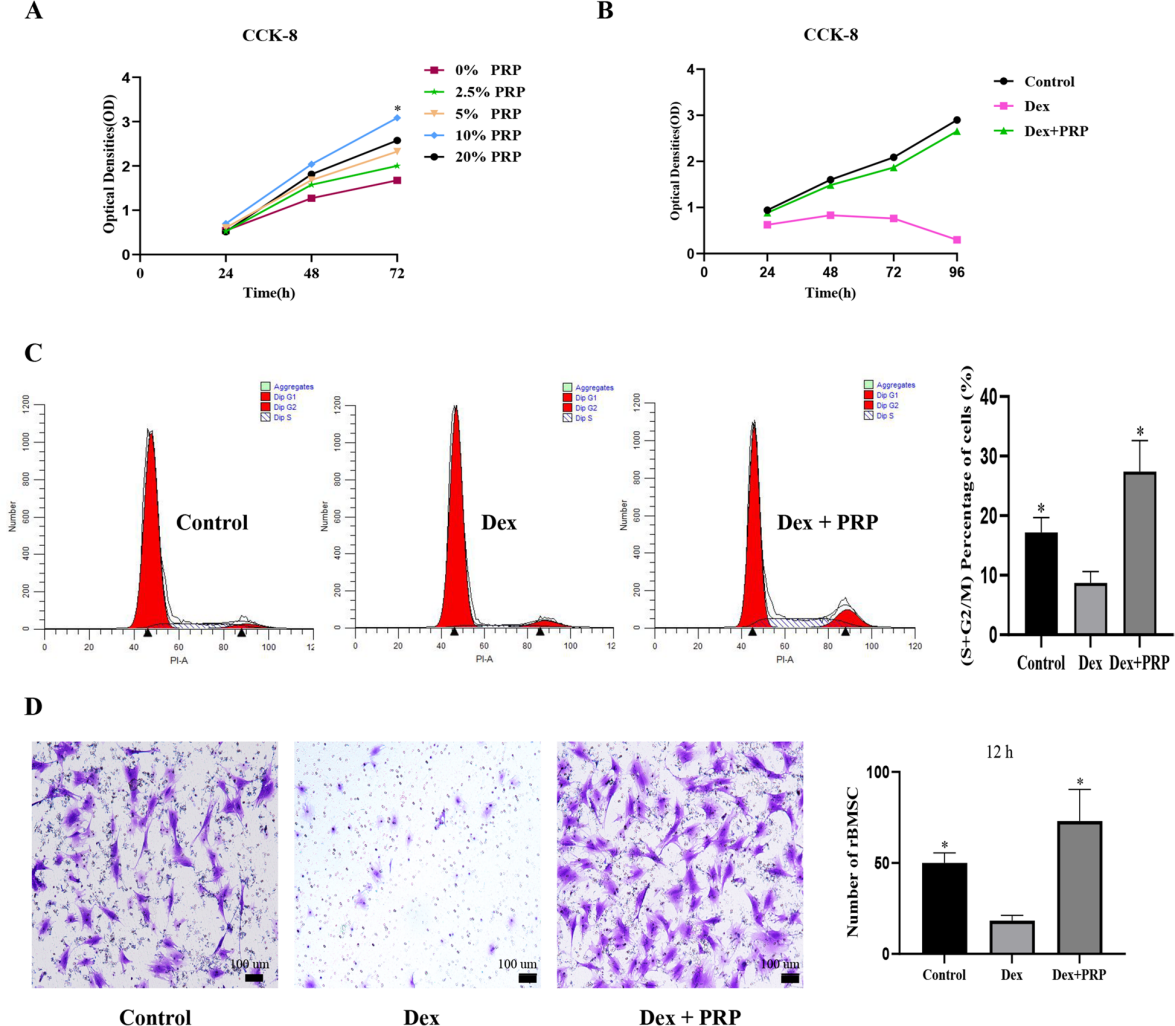
PRP alleviated the inhibitory effects of Dex on the proliferation and migration of BMSCs. **A** Effect of different concentrations of PRP on cell proliferation. The treatment of BMSCs with PRP caused maximum extent proliferation. **B** After treatment of Dex and Dex supplemented with PRP, the proliferation of BMSCs was detected by CCK-8. **C** Flow cytometry analysis of cell cycle after treatment with PRP for 24 h. The number ratio of cells in the (S + G_2_/M) phase was significantly increased in the Dex + PRP group compared with that in the Dex group, demonstrating the state of cell proliferation. **D** Transwell assay was used to detect the migration capacity changes of BMSCs after treatment. (*p < 0.05 vs. Dex group)

**Fig. 2:**
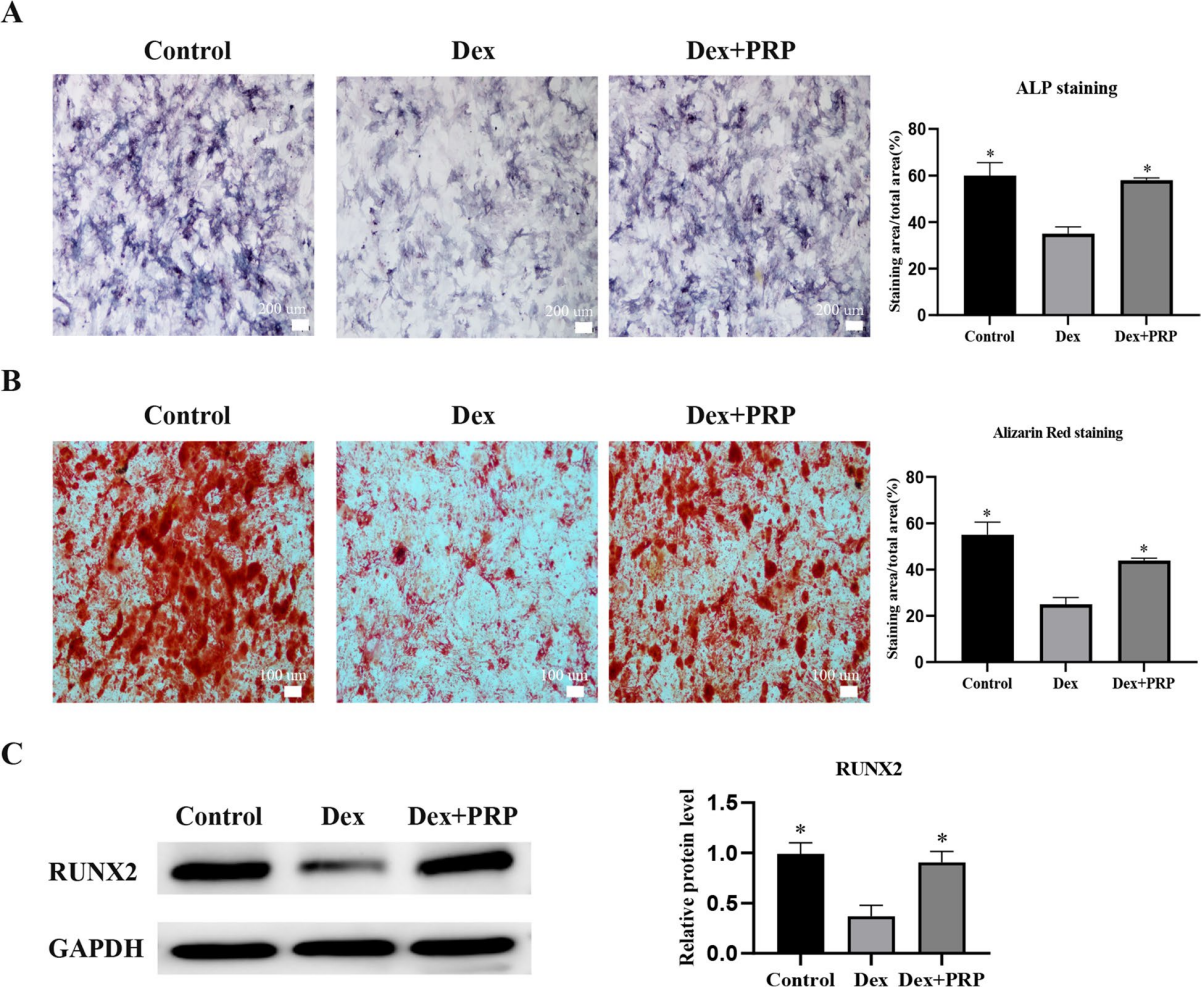
PRP resisted the apoptosis induced by Dex in BMSCs. **A** TUNEL assay evaluated cell apoptosis. **B** Western blot analysis shows the expression for Bcl-2 and Caspase-3 apoptosis-related protein in BMSCs exposed to Dex and co-treated with PRP. GAPDH served as an endogenous control. (*p < 0.05 vs. Dex group)

The platelet concentration was (2875 ± 236) × 10^9^ / L, about four times that of the whole blood (650 ± 89) × 10^9^ / L. After the PRP purification process, the presence of white blood cells in the PRP sample (0.007 ± 0.0065) × 10^9^/ L was drastically reduced, as the whole blood sample showed a raw white blood cell count of 7.2 × 10^9^ /L. Therefore, the samples obtained should be considered as leucocyte-poor platelet-rich plasma (P-PRP). The concentration of TGF-β was (139 ± 21.3) μg / L. As shown in Fig. 1A, compared with no addition of PRP, PRP significantly increased the growth of BMSCs. The proliferation of BMSC at 20% PRP is weaker than that at 10% PRP. From the above result, we can conclude that the treatment of BMSCs with 10% PRP caused maximum extent proliferation. Accordingly, all further experiments were conducted with 10% PRP. The CCK-8 assay and the transwell migration assay showed that BMSCs proliferation and migration capacities were significantly suppressed by Dex, while this inhibition was antagonized by PRP (Fig. 1B and D). After 24 h treatment with PRP, the number ratio of cells in the (S + G_2_/M) phase was significantly increased compared with that in the Dex group (Fig. 1C). In addition, compared to the control group, 48 h of Dex treatment significantly promoted the apoptosis of BMSCs, and this effect could be ameliorated after PRP treatment (Fig. 2A). We further detected the expression of apoptosis-related proteins by Western Blot. After PRP treatment, the apoptosis marker Caspase-3, was significantly down-regulated while the expression of anti-apoptosis marker Bcl-2,was significantly up-regulated (Fig. 2B).

### PRP alleviated the inhibitory effects of Dex on osteogenic differentiation of BMSCs

**Fig. 3:**
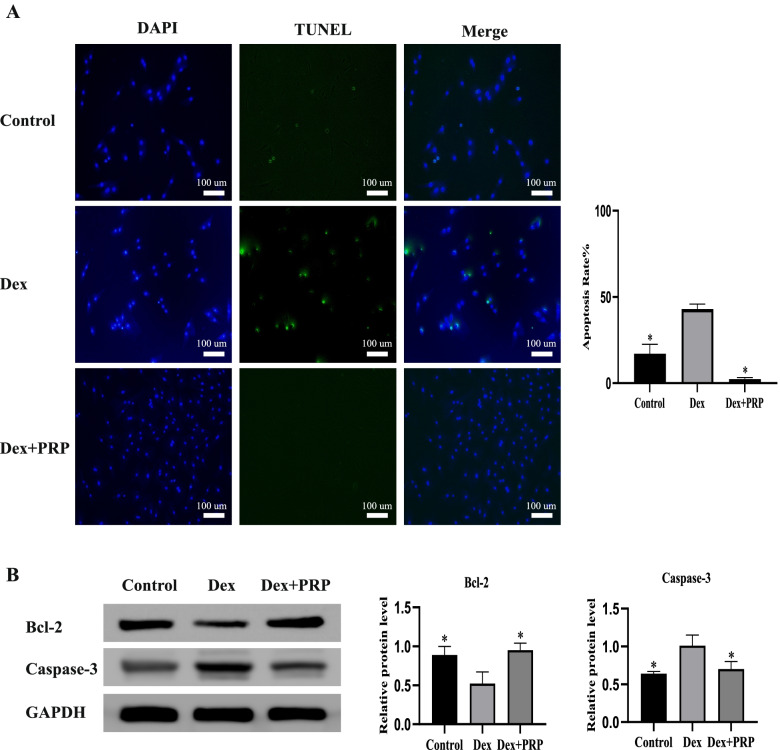
PRP alleviated the inhibitory effects of Dex on osteogenic differentiation of BMSCs. **A** and **B** After induction of osteogenic differentiation, ALP activity and calcium deposition were detected by ALP staining (7 d) and alizarin red staining (14 d), respectively, and quantitative analysis was performed. **C** Western blot analysis shows the expression for RUNX2 osteogenesis-related protein in BMSCs exposed to Dex and co-treated with PRP. GAPDH served as an endogenous control. (*p < 0.05 vs. Dex group)

ALP staining and alizarin red staining results showed that Dex could inhibit the process of osteogenesis while PRP treatment mitigated this effect of Dex (Fig. 3A and B). The protein level the osteogenic marker Runx2 was significantly up-regulated in the Dex + PRP group.

## Discussion

In recent years, with the wide application of glucocorticoid in clinical practice, GIONFH has become a major cause of non-traumatic necrosis of the femoral head [1, 2]. Although there have been many treatment attempts to prevent the progression of GIONFH, the potential effective strategy is still under investigation [2]. In this study, we found PRP could enhance the proliferation and anti-apoptotic activities of Dex-induced BMSCs. Furthermore, Dex-induced suppression of cell osteogenic differentiation was rescued by PRP *in vitro*.

Previous studies have clearly shown that activated PRP promotes the proliferation of multiple MSCs lines *in vitro* in a concentration-dependent manner [22–25]. Consistent with these findings, our research showed that higher concentrations of activated PRP had a stronger effect on BMSCs. In our study, 10% PRP was found as the optimal concentration of BMSCs proliferation, which was similar to the results of previous studies [23, 26]. Interestingly, however, the effect of increased PRP concentration (20% PRP) did not show similar or enhance BMSC proliferation effect, indicating that there is an optimal dosage of PRP. Further research may be needed to study whether PRP can improve the activity of BMSCs induced by high-dose glucocorticoids.

In this study, Dex reduced the proportion of S + G2/M (proliferation index, PrI) phase cells and increased cell apoptosis, while PRP could reverse the glucocorticoid-induced inhibition. S + G2M phase represents the percentage of proliferating cells in the population, reflecting the state of cell proliferation to a certain extent [20]. The apoptosis rate and the levels of apoptosis-related protein Caspase-3 increased after Dex-induced, while anti-apoptosis-related protein Bcl-2 was decreased. PRP interfered with these effects of Dex. These results indicated that the mechanism by which PRP improved the proliferation of BMSCs and inhibited apoptosis may be related to the growth factors released by PRP to increase the DNA replication of BMSCs. In addition, the migration capacity is essential for the exertion of BMSCs functions. In our study, we confirmed that PRP alleviated the inhibitory effects of Dex on the migration of BMSCs *in vitro* Whether PRP can enhance the migration of precursor cells and successful adhesion and colonization is the key to improving the ability of bone regeneration [24, 27].

In addition, it is worth mentioning that the leucocyte-poor platelet-rich plasma (P-PRP) was used in our study. P-PRP has been shown to be more suitable for bone regeneration [28]. The platelet concentration of PRP in this study was 4 times higher than that of the basal platelet concentration of the whole blood, in which the concentration of TGF-β was high. It has been shown that TGF-β played an important role in osteogenic differentiation. TGF-β can regulate the osteoblastic differentiation of mesenchymal stem cells in the early stage and regulate the collagen secretion and calcium deposition of osteoblasts in the late stage [29]. ALP is an early marker of osteoblastic differentiation, and alizarin red staining calcium nodules are a marker of middle and late osteoblastic differentiation and functional maturation [12]. In this experiment, ALP staining and alizarin red staining showed that PRP alleviated the inhibitory effects of Dex on osteogenic differentiation of BMSCs. The mechanism may be related to PRP being rich in TGF-β. However, further researches are needed to fully clarify the mechanisms involved in this process.

In the present study, we investigated whether PRP improved the activity of BMSCs induced by Dex. In addition, we are conducting a preliminary study on the treatment of ONFH with intraarticular PRP injection. We believe that this basic study could provide partial mechanisms for further clinical practice for the treatment of ONFH with PRP.

This study has several limitations. First, the components of PRP are relatively complex, and its specific mechanism needs further study. Second, there was only an *in vitro* study. The mechanism of PRP on ONFH *in vivo* was still unclear, since the *in vitro* environment could not simulate the intra-articular physiological conditions. Furthermore, the results of *in vitro* studies can not be directly transferred to a clinical application, especially for the dosage consideration. But through our *vitro* study, we can infer that PRP had a protective effect on ONFH. In future research, we may further establish ONFH animal models for mechanism research.

## Conclusion

In conclusion, our studies showed that PRP significantly protected BMSCs from Dex-induced apoptosis, promoted BMSCs proliferation, migration, and osteogenic differentiation. This study provides a promising strategy for the treatment of GIONFH with PRP. It also indicates that PRP is a promising drug that may be used to prevent ONFH, which also lays the foundation for the application of PRP in other musculoskeletal diseases.

## Supplementary Information

**Additional file 1.** Western blot original images.

## Data Availability

The data analyzed during the current study are available from the corresponding author on reasonable request.

## Abbreviations

GIONFH: Glucocorticoid-induced osteonecrosis of the femoral head
PRP: platelet-rich plasma
P-PRP: leucocyte-poor platelet-rich plasma
BMSCs: bone marrow mesenchymal stem cells
Dex: dexamethasone
ALP: alkaline phosphatase
ALR: alizarin red staining
