# Correction to: Therapeutic effect of platelet-rich plasma on glucocorticoid-induced rat bone marrow mesenchymal stem cells *in vitro*

**DOI:** 10.1186/s12891-022-05353-2

**Published:** 2022-05-06

**Authors:** Yanxue Wang, Shuo Luan, Ze Yuan, Caina Lin, Shengnuo Fan, Shaoling Wang, Chao Ma, Shaoling Wu

**Affiliations:** Department of Rehabilitation Medicine, Sun Yat-sen Memorial Hospital, Sun Yat-sen University, Guangzhou, 510030 Guangdong China

**Correction to: BMC Musculoskelet Disord 23, 151 (2022)**

**https://doi.org/10.1186/s12891-022-05094-2**

Following the publication of the original article [1] the authors reported that legend order of Figs. 2 and 3 was wrongly reversed.

The original article [1] has been updated.

Below are Figs. 2 and 3 with the correct legends.

**Fig. 2:**
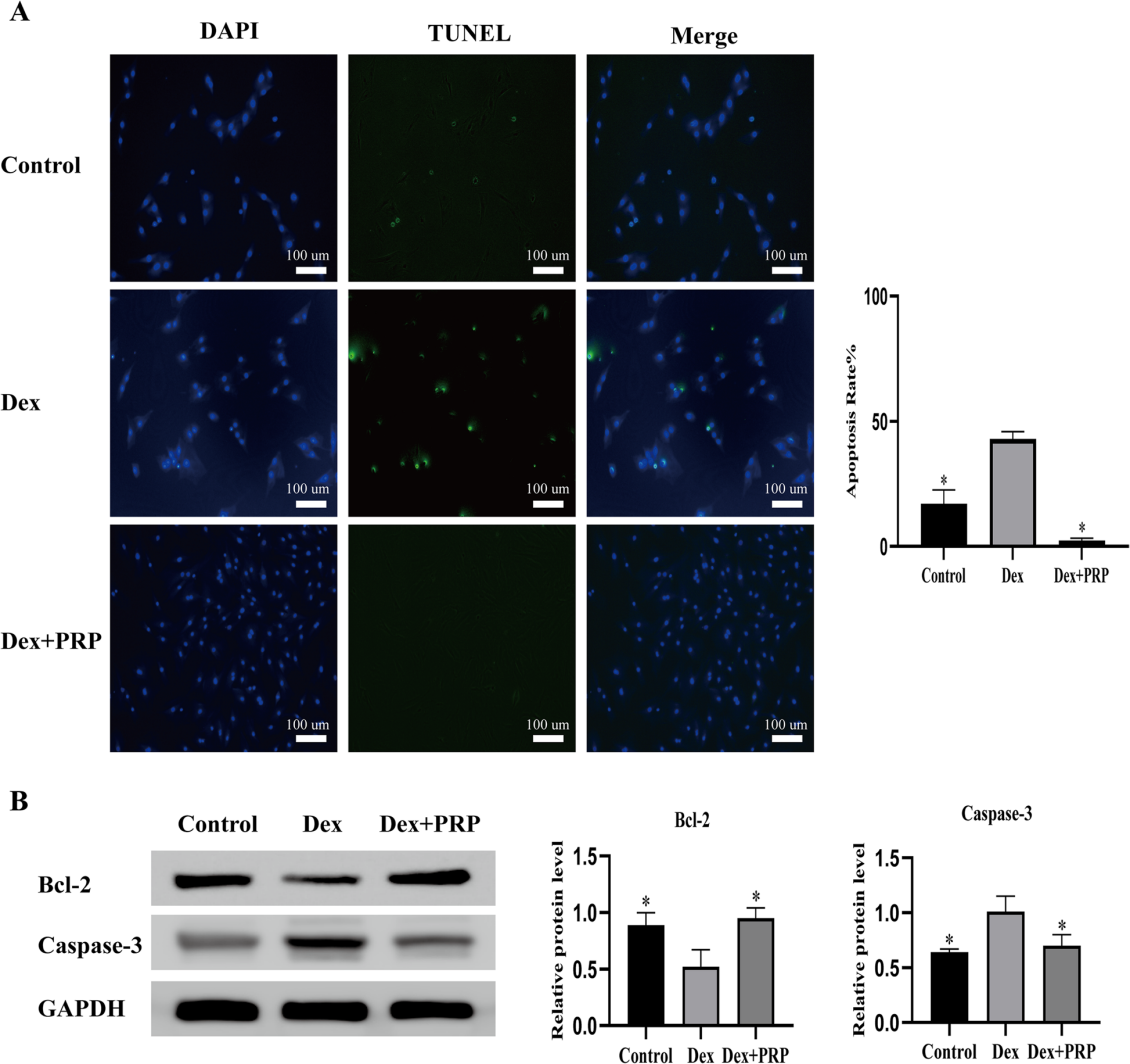
PRP resisted the apoptosis induced by Dex in BMSCs. **A** TUNEL assay evaluated cell apoptosis. **B** Western blot analysis shows the expression for Bcl-2 and Caspase-3 apoptosis-related protein in BMSCs exposed to Dex and co-treated with PRP. GAPDH served as an endogenous control. (*p < 0.05 vs. Dex group)

**Fig. 3:**
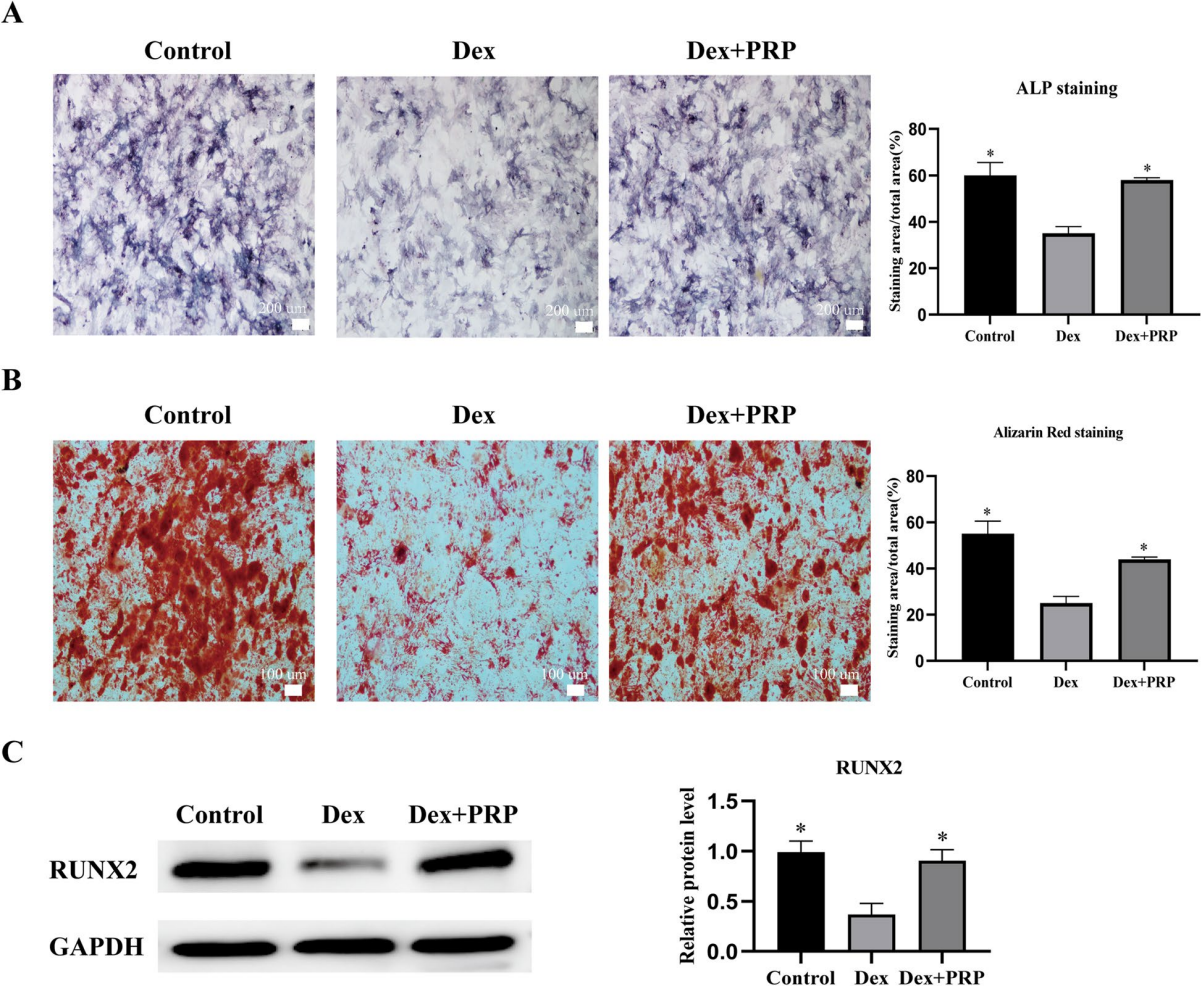
PRP alleviated the inhibitory effects of Dex on osteogenic differentiation of BMSCs. **A** and **B** After induction of osteogenic differentiation, ALP activity and calcium deposition were detected by ALP staining (7 d) and alizarin red staining (14 d), respectively, and quantitative analysis was performed. **C** Western blot analysis shows the expression for RUNX2 osteogenesis-related protein in BMSCs exposed to Dex and co-treated with PRP. GAPDH served as an endogenous control. (*p < 0.05 vs. Dex group)
