# Supplementary figures and images for: Therapeutic effect of platelet-rich plasma on glucocorticoid-induced rat bone marrow mesenchymal stem cells in vitro
Source: BMC Musculoskelet Disord. 2022 Feb 15;23:151. doi: 10.1186/s12891-022-05094-2 (PMC8845312; doi:10.1186/s12891-022-05094-2)

# Western Blot Original Images

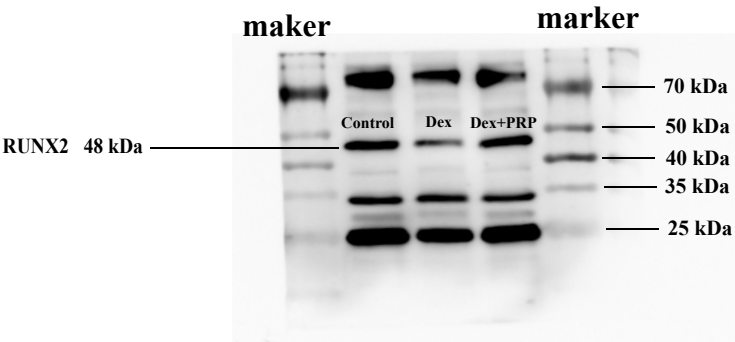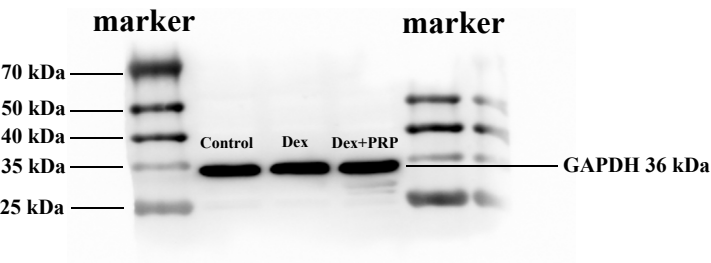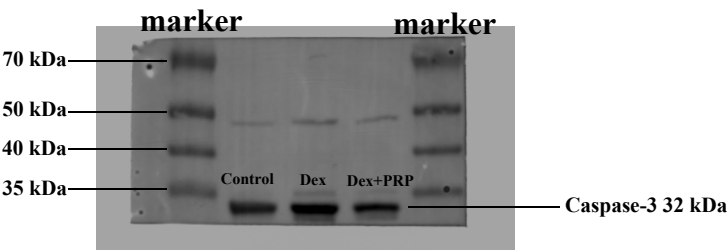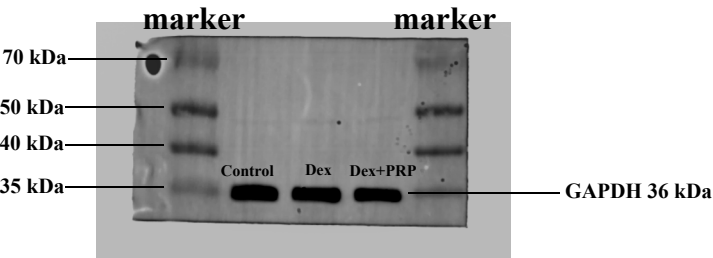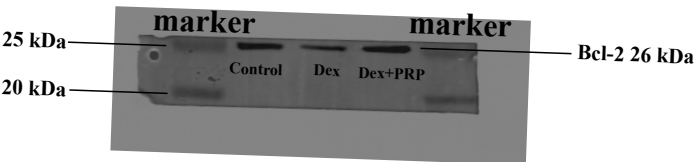

Supplement: Supplementary file 1 — Additional file 1. Western blot original images. [file 12891_2022_5094_MOESM1_ESM.pdf]
